# Impact of protein supply on the productive performance of growing lambs drinking natural saline water and fed low-quality forage under semi-arid conditions

**DOI:** 10.1007/s11250-023-03462-1

**Published:** 2023-02-01

**Authors:** Amr A. Gabr, Mona E. Farag, Gamal F. Shahin, Essam M. El-Kotamy

**Affiliations:** 1 Department of Animal Production, Faculty of Agriculture, Mansoura University, Mansoura, 35516 Egypt; 2 Department of Animal Nutrition Research, Animal Production Research Institute, Agricultural Research Center, Dokki, 12619 Giza Egypt

**Keywords:** Saline water, Protein supplementation, Barki sheep, Growth performance, Feed intake, Animal management

## Abstract

Consuming saline water causes animals salinity stress, which leads to many adapting metabolic changes that could negatively affect its performance and the quality of the derived products. Therefore, this study aimed to evaluate the impact of increasing diet protein level on the productive performance of growing lambs drinking natural saline water in Egyptian semi-arid region. Twenty-four growing Barki lambs (4–5 months old) with an initial body weight of 20.7 ± 0.25 kg were randomly distributed into four similar groups for 150 days. Two diets were formulated: low protein and high protein levels (concentrate feed mixture containing 14% and 20% crude protein (CP) on dry matter basis, respectively). Within each level of CP, natural saline water was represented by low saline (LS) and high saline (HS) water, containing 658 and 2100 mg/L of total dissolved solids, respectively. Results showed that the HS water increased (*p* = 0.02) water intake by about 18% and had adverse effect (*p* < 0.001) on dry matter intake, nutrient digestibility, and growth performance. The ruminal pH values, total volatile fatty acids, and ammonia–N concentrations were not affected by drinking the HS water. However, the protein supplementation enhanced the HS lambs’ nutrients digestion and showed greater growth performance. The HS water decreased (*p* < 0.001) the serum concentrations of alanine aminotransferase (ALT) and aspartate aminotransferase (AST) and increased (*p* = 0.03) the urea-N by about 9%. The protein supplementation amended the serum ALT and AST concentrations of HS lambs. It is concluded that the dietary protein supply was affective sustainable management strategy against the deleterious effect of drinking high saline water on growing lambs.

## Introduction

Global climate changes increase the risk of soil and water salinization, especially in arid and semi-arid regions, imposing possible health risks on livestock (Enke et al. 2022) and creating new threats for animal production. Moreover, the amounts of available drinking water for farm animals may become limited especially during the long dry season. Consequently, under such conditions, the livestock watering becomes expensive and should be considered and computed in the economical production cost. On the other hand, animals could likely suffer, to a certain level, from meeting their water intake and nutritional requirements. This should lead animals to minimize water losses and maintain essential physiological processes by activating several water saving mechanisms (Dickhoefer et al. 2021).

Although small ruminants can be gradually adapted to drink saline water (Costa et al. 2021), their ability to tolerate saline water depends on the salinity level and the type of salt minerals in water as well as the animal species (Abdelsattar et al. 2020). In general, numerous studies were conducted often with additions of minerals sources to simulate natural sources of saline water, while very few studies have used actual natural sources of saline drinking water (Yirga et al. 2018). However, consuming high saline water causes small ruminant salinity stress, which leads to many adapting metabolic changes that could negatively affect its performance (Hussein et al. 2020) and the quality of the derived products (Costa et al. 2021). Furthermore, several studies indicated that high saline water negatively affected the small ruminants’ food consumption and digestibility, decreased the growth performance, and had adverse effect on some physiological and biochemical parameters (López et al. 2017; Araújo et al. 2019; Abdelsattar et al. 2020; Costa et al. 2021; Noureddine et al. 2022; Thiet et al. 2022). The effects of high saline water constituting a major constraint could affect the efficiency and sustainability of livestock systems (Noureddine et al. 2022).

The small ruminant is actively livestock breeds in the world’s arid and semi-arid varied regions. The Barki sheep breed is one of the main sheep breeds in Egypt, and it is well adapted to produce a considerable production under the harsh desert conditions in the Mediterranean zone (Abousoliman et al. 2020). Within the Egyptian reclamation strategy, groundwater that has high salinity will be the main source of used water in cultivated arid and semi-arid regions (Abdelsattar et al. 2020). Sheep production under such conditions faces multiple challenges, such as water scarcity, high feeding costs, and feeding systems based on low-quality forages (El-Zaiat et al. 2020).

The dietary protein supplementation can play an effective role as one of the most popular ruminant dietary supplements used to improve forage intake, ruminal digestibility, and performance. The pattern of response to protein supplementation can be altered by the ingestion of large amounts of high saline water when ruminants are fed low-quality forages (López et al. 2017). Accordingly, using protein supplementation as sustainable development management strategy for raising small ruminants on saline water needs understanding some necessary information of the interaction between protein supplementation and saline water on the animal drinking and eating behaviors, the low-quality forage consumption and utilization, the animal physiological responses, and the maximum concentration of water salinity the animal can tolerate. This information is crucial to improve the predictability of animal response to the protein supplementation. However, very few comparative studies have investigated the protein supplementation and natural saline water interaction for productive performance in growing lambs. The existence of an interaction between supplemental protein and high saline water that affect the response of sheep to low-quality forage utilization was confirmed (López et al. 2017).

However, it is fundamental to know the impact of protein supplementation on the water salinity tolerance of growing lambs raised in semi-arid regions and fed diets contained of low-quality forage for better use of saline waters without causing deleterious effect on the production efficiency. Therefore, the present study evaluated the impact of dietary protein supplementation on feed efficiency, some ruminal and blood parameters, and productive performance of growing Barki lambs fed rations containing low-quality forage when drinking natural low or high saline water in Egyptian semi-arid region.

## Materials and methods

### Animal diet and management

The experiment was conducted at private farm located in Tanbul, Wadi El Natrun, Beheira governorate, Egypt (30°13′48.1″N 30°26′18.6″E). The study was respectfully carried out in the manner of the stated ethics and animal rights (DRC) in accordance with the European Union Directive Regulations (2010/63/EU) regarding the protection of animals used for experimental and other scientific purposes. The study was approved by the Animal Production Research Institute, Agricultural Research Center, Egypt (protocol code 162,429).

Twenty-four growing male Barki lambs were used, with an average live body weight of 20.7 ± 0.25 kg and average age of 4 to 5 months. Animals were randomly distributed into four similar groups (6 lambs/group); each group was housed in separate semi-opened pens subdivided into individual cages of 1.5 m^2^ per lamb throughout the experimental period of 150 days. Two basal diets were formulated with two different levels of concentrate feed mixture (CFM) crude protein (CP) (Tables 1 and 2): low protein level (LP) (14% CP on DM basis) and high protein level (HP) (20% CP on DM basis). Both diets were formulated with the same ingredients and additives. Within each level of CP, all the lambs were fed a diet of low-quality forage (rations were 40% CFM, 50% rice straw, and 10% berseem hay). The experimental rations were formulated to meet the growing lambs’ requirements (National Research Council, 1985). Water saline levels were represented by natural sources (water wells) of low saline (LS) and high saline (HS) water, containing 658 and 2100 mg/L of total dissolved solids (TDS), respectively (Table 3). The treatment structure was a 2 × 2 factorial, which resulted from the combination of two levels of water salinity and two levels of crude protein.

**Table 1:** Ingredients of concentrate feed mixture of the experimental diets

Ingredients	Concentrate feed mixture CFM	
	Low crude protein	High crude protein
Yellow corn	37	20
Wheat bran	30	35
Undecortecated cotton meal	12	7.0
Undecortecatedi sunflower meal	10	7.0
Soyabean meal, 44% CP	2.0	22
Molasses	5.0	5.0
limestone	3.0	3.0
Vitamin–mineral premix*	1.0	1.0

^*^Ingredients of vitamin–mineral premix contained in each kilogram of feed (DM basis): Co, 5 mg; Cu, 5 mg; Fe, 400 mg; Zn, 125 mg; Mn, 90 mg; I, 0.01%; K, 1%; Na, 0.60%; Mg, 0.15%; S, 0.40%; Se, 0.02%; vitamin A, 55,000 IU; vitamin D, 5,600 IU; vitamin E, 350 IU; vitamin B1, 15 mg; vitamin B2, 35 mg; vitamin B6, 25 mg; vitamin C, 100 mg; and pantothenate 75 mg

**Table 2:** Chemical composition (%) of the experimental rations

	Concentrate feed mixture		BH	RS	Experimental rations	
	LP*	HP			LP	HP
Dry matter	89.9	99.1	88.7	92.8	91.2	94.9
On dry matter basis;						
Organic matter	92.0	91.1	87.7	80.0	85.5	85.2
Crude protein	14.0	20.0	11.9	2.76	8.17	10.6
Ether extract	3.56	3.06	2.43	1.36	2.35	2.15
Crude fiber	9.56	9.28	24.1	37.9	25.2	25.1
Nitrogen-free extract	64.8	58.7	49.3	38.0	49.8	47.4
Ash	8.05	8.92	12.3	20.0	14.5	14.8

^*^LP, low crude protein; *HP*, high crude protein; *BH*, berseem hay; and *RS*, rice straw

**Table 3:** Total dissolved solids and mineral content (mg/L) of drinking water

	Drinking water	
	Low saline water	High saline water
pH	7.31	7.98
Calcium	63.0	379
Magnesium	11.4	39.0
Sodium	185	641
Potassium	2.51	11.8
Bicarbonate	246	566
Total dissolved solids (TDS)	658	2100

### Feed intakes and growth performance

The concentrate feed mixture was weighted for each group and offered twice daily at 7:00 and 16:00 h, while roughage was offered at 8:00 and 17:00 h in two equal amounts. The offered CFM and roughages were recorded daily, and the refused quantities of each ingredient were recorded once weekly. At feeding time, the drinking water tanks were refilled, and lambs had continuous access to water. The water consumption was measured by subtracting refused water from offered water. To calculate the average daily gain (ADG, g/day), lambs were fasted for 18–20 h and individually weighed at the onset of the study and biweekly thereafter. Feed allowance was adjusted biweekly according to the change in lambs body weight to calculated feed efficiency.

### Feed, water, and feces samples

The samples of concentrate, roughage, and feces were collected to estimate the apparent nutrient digestibility coefficients. At the last 3 weeks of the experiment period of 150 days, twelve lambs (3 lambs from each group) were randomly selected and placed in metabolic cages (0.54 × 1.5 m) to determine digestibility coefficients and nutritive values of experimental rations. After an acclimation period of 2 weeks, each lamb feces were collected twice daily at 07:00 and 15:00 h for one more week. The feces of each lamb were weighted and stored (10% of daily output) at − 20 ^0^C. Fecal samples were dried in an oven at 60 ^0^C for 72 h, mill-ground, and kept for further chemical analyses. The feed and fecal samples were analyzed for DM (at 100 ^0^C in the oven for 24 h), CP (Kjeldahl procedure; 6.25 × N), ash, and ethereal extract (EE) using procedures established by AOAC (2019). Fiber contents (CF) were analyzed using Van Soest’s (1991) ANKOM 2000 procedure. The nitrogen-free extract (NFE) was calculated using the following formula: NFE = ((organic matter (OM) − (EE + CP + CF)). Digestion coefficients of all nutrients and feeding values were calculated for each experimental group. Feeding values, including the total digestible nutrient (TDN) and digestible crude protein (DCP), were calculated according to the National Research Council (2001). The samples of drinking water were weekly collected throughout the experimental period of 150 days, conditioned in labeled plastic bags and frozen until they were analyzed. The water analyses of calcium, sodium, and potassium were performed using the flame photometry method, while magnesium and bicarbonate were determined by titration using ethylenediaminetetraacetic acid disodium salt and sulfuric acid, respectively (Estefan et al. 2013). Total dissolved solids were measured using TDS meter (JENCO, 3173COND, USA).

### Rumen parameters

On the last day of experimental period, at 3 h post the morning feeding, the ruminal fluid was collected by flexible stomach tube from the lambs of the digestibility trial. After discarding 50 mL of the initial ruminal fluid, 100 mL of ruminal fluid was collected and filtered through three layers of cheesecloth. Ruminal fluid pH was directly measured with digital pH meter (Adwa, AD11, Romania). Sub-samples of 5 mL ruminal fluid were preserved in dry clean glass bottles with 5 mL of 0.2 M HCl for ammonia–nitrogen analysis (NH3-N) (AOAC 2005). For total volatile fatty acids’ (VFAs) analysis, a 0.8 mL of ruminal fluid was mixed with 0.2 mL of a solution containing 250 g of metaphosphoric acid/L and centrifuged at 15,000 rpm for 20 min at 4 °C (K1015 Micro Prime; Centurion Scientific Ltd, Stoughton, Chichester, UK). The VFAs’ concentration was measured using gas chromatography (Thermo fisher scientific, Inc., TRACE1300, Rodano, Milan, Italy) fitted with an AS3800 autosampler and equipped with a capillary column HP-FFAP (19091F-112; 0.320 mm outer diameter, 0.50 µm inner diameter, and 25 m length; J&W Agilent Technologies Inc., Palo Alto, California, USA).

### Blood sampling and serum biochemical analysis

Blood samples (10 mL) were withdrawn from jugular vein of lambs at 3 h after morning feeding. Blood was clotted at room temperature for 20 min and centrifuged at 3000 rpm for 10 min to obtain the serum. Serum samples were immediately separated, transported, and stored at − 20 ^0^C until the subsequent analysis. Blood serum samples were analyzed for total protein, albumin, alanine aminotransferase (ALT), aspartate aminotransferase (AST), urea, and creatinine using commercial test kits provided by (Spectrum Diagnostics, Egypt) and a spectrophotometer (Clinical Chemistry Analyzer ERBA CHEM 7, ERBA, Mannheim, Germany). The serum globulin concentration was calculated by subtracting the obtained albumin value from the total protein value.

### Statistical analysis

The data were analyzed with general linear model (GLM) procedure of SAS version 9.3 (SAS Institute Inc. Cary, NC, USA) by using the following statistical model, Yijk = μ + Pi + Wj + (T × W)ij + eijk, where Yijk is the observation from lamb; μ is the overall mean; Pi is the experimental dietary treatment effect; Wj is the salinity water effect; (P × W)ij is the interaction between dietary treatment and water treatment; and eijk is the residual error. Least square means and their standard error are described. For all data, the lamb was the experimental unit. An integrated Tukey’s test was used to evaluate differences between groups at the 5% probability level.

## Results

### Effect on feed and water intake

The high saline water (2100 mg TDS/L) increased (*p* = 0.02) the lambs’ daily water intake by about 18% and decreased the low-quality roughage, dry mater, TDN, and DCP intakes than LS lambs’ groups that fed the same CP level (Table 4). However, the protein supplementation did not affect the lambs’ water and feed intakes. Almost similar values of daily DM intakes were observed in HS lambs’ groups, while the protein supplementation increased (*p* < 0.001) the daily TDN and DCP intakes of HS lambs (7.4% and 30%, respectively) than HS lambs fed the LP ration. There was no interaction between water salinity level and CP level.

**Table 4:** Effects of protein supplementation and water salinity on feed utilization efficiency and water intake of growing Barki lambs

	LP*		HP		SEM	*p* values		
	LS	HS	LS	HS		Protein	Water	P*W
Av. daily feed intake, kg								
Concentrate feed mixture	0.62	0.57	0.63	0.56	0.02	0.85	0.07	0.73
Barssim hay	0.16	0.14	0.16	0.14	0.01	0.96	0.61	0.93
Rice strow	0.77^a^	0.71^b^	0.79^a^	0.70^b^	0.02	0.80	0.03	0.65
Total dry matter intake	1.55^a^	1.42^b^	1.59^a^	1.41^b^	0.03	0.63	< 0.001	0.39
Av. daily feed unit intake, kg								
Dry matter	1.45^a^	1.35^b^	1.47^a^	1.36^b^	0.02	0.55	0.01	0.89
Total digestible nutrient	0.87^b^	0.75^d^	0.90^a^	0.81^c^	0.02	0.18	0.01	0.66
Digestible crude protein	0.08^c^	0.07^d^	0.11^a^	0.10^b^	0.004	< 0.001	< 0.001	0.98
Av. daily water intake								
Water intake, L	3.73^b^	4.55^a^	3.98^b^	4.89^a^	0.19	0.33	0.02	0.88
Water intake, ml/kg body weight	126^b^	164^a^	127^b^	165^a^	5.73	0.11	< 0.001	0.67
Water intake, ml/kg dry matter	2.57^b^	3.38^a^	2.70^b^	3.61^a^	0.18	0.56	0.02	0.87

^*^LP, low crude protein ration (containing concentrate feed mixture CFM of 14% CP); *HP*, high crude protein ration (containing CFM of 20% CP); *LS*, low saline water (658 mg/L total dissolved solids); and *HS*, high saline water (2100 mg/L total dissolved solids). ^a,b,c,d^within a row, values with different superscript letters mean there were significant differences (*p* < 0.05)

### Effect on nutrient digestibility, feeding values, and rumen parameters

The increase in the water salinity from 658 to 2100 mg TDS/L affected (*p* < 0.05) the digestion coefficient of DM, OM, CP, CF, and EE of HS lambs compared to LS lambs of the same rations, but it had no effect on NFE digestibility (Table 5). The protein supplementation improved (*p* < 0.01) HS lambs’ digestion of DM, OM, and CP by 7%, 4%, and 5.5%, respectively, compared with those of HS lambs fed the LP ration. Likewise, as a result of protein supplementation for HS lambs, the feeding values as TDN and DCP were higher (*p* < 0.05) than HS lambs of LP ration by 5.9% and 23.5%, respectively, without significant differences with the LS lambs fed HP ration (Table 5).

**Table 5:** Effects of protein supplementation and water salinity on nutrient digestion coefficients, feeding values, and ruminal parameters of growing Barki lambs

	LP*		HP		SEM	*p* values		
	LS	HS	LS	HS		Protein	Water	P*W
Nutrient digestibility, %								
Dry matter	63.8^b^	60.0^c^	68.4^a^	64.6^b^	0.93	< 0.001	< 0.001	0.98
Organic matter	64.2^b^	62.5^c^	69.3^a^	65.2^b^	0.80	< 0.001	0.001	0.07
Crude protein	66.7^a^	63.7^b^	68.2^a^	67.4^a^	0.57	< 0.01	0.01	0.10
Crude fiber	55.8	53.7	56.4	54.4	0.41	0.29	0.01	0.94
Ether extract	70.5	67.4	71.8	68.1	0.59	0.12	< 0.001	0.61
Nitrogen-free extract	73.8	68.4	67.5	75.0	1.02	0.83	0.11	< 0.001
Feeding values, %								
Total digestible nutrient	60.1^a^	56.1^b^	61.2^a^	59.6^a^	0.62	< 0.01	< 0.01	0.06
Digestible crude protein	5.46^b^	5.20^b^	7.21^a^	6.80^a^	0.36	0.02	0.578	0.90
Ruminal parameters								
pH values	6.89	6.70	6.58	6.61	0.25	0.74	0.89	0.85
Total VFA's, meq/100 ml	11.4	11.5	12.0	11.8	0.25	0.52	0.95	0.80
NH3–N, mg/100 ml	11.6^b^	13.1^b^	18.1^a^	16.6^a^	0.81	< 0.001	0.99	0.03

^*^LP, low crude protein ration (containing concentrate feed mixture CFM of 14% CP); *HP*, high crude protein ration (containing CFM of 20% CP); *LS*, low saline water (658 mg/L total dissolved solids); and *HS*, high saline water (2100 mg/L total dissolved solids). ^a,b,c,d^within a row, values with different superscript letters mean there were significant differences (*p* < 0.05)

Ruminal pH values and total VFA’s concentrations were not affected by drinking high saline water and protein supplementation (Table 5). High saline water tended to do so as well for ruminal ammonia–N concentration since no significant differences were recorded between groups that fed the same CP ration, but the values increased (*p* < 0.001) as response to protein supplementation by about 36% and 21% for LS and HS water, respectively.

### Effect on blood serum parameters

The results in Table 6 summarized some blood biochemical variables in Barki lambs as affected by protein supplementation and drinking saline water. Saline water had no effect on the lambs’ blood concentrations of total protein, albumin, globulin, and albumin/globulin ratio, whereas blood total protein increased as lambs supplemented with protein. The AST and ALT concentrations tended to be affected by water salinity. AST and ALT were decreased (*p* < 0.001) in the HS lambs compared to LS lambs of LP and HP groups by about 12.8%, 18.6%, and 12%, 16.6%, respectively, while protein supplementation decreased (*p* < 0.05) the effect on the HS lambs. Conversely, the HS lambs showed higher urea-N concentration than LS lambs of LP and HP groups by about 9.4% and 9.7%, respectively. The concentration of creatinine was tended to be similar among tested groups.

**Table 6:** Effects of protein supplementation and water salinity on some blood serum parameters of growing Barki lambs

	LP*		HP		SEM	p values		
	LS	HS	LS	HS		Protein	Water	P*W
Serum protein, g/dL								
Total protein	6.36	6.27	6.83	6.60	0.26	0.51	0.79	0.91
Albumin	3.84	3.81	3.97	3.91	0.25	0.85	0.94	0.98
Globulin	2.52	2.46	2.86	2.69	0.25	0.64	0.85	0.93
Albumin/globulin ratio	1.52	1.55	1.39	1.45	0.06	0.31	0.70	0.87
Liver function, IU/L								
Aspartate aminotransferase	39.1^a^	34.1^c^	41.1^a^	36.2^b^	0.84	0.01	< 0.001	0.95
Alanine aminotransferase	31.1^a^	25.3^c^	32.6^a^	27.2^b^	0.92	0.02	< 0.001	0.74
Kidney function, mg/dL								
Urea-N	12.6^b^	13.9^a^	13.1^b^	14.5^a^	0.78	0.11	0.03	0.56
Creatinine	1.20	1.08	1.22	1.11	0.25	0.97	0.85	0.99

^*^LP, low crude protein ration (containing concentrate feed mixture CFM of 14% CP); *HP*, high crude protein ration (containing CFM of 20% CP); *LS*, low saline water (658 mg/L total dissolved solids); and *HS*, high saline water (2100 mg/L total dissolved solids). ^a,b,c^ within a row, values with different superscript letters mean there were significant differences (*p* < 0.05)

### Effect on growth performance

The effects of experimental diets on the productive performance of lambs are shown in Table 7. The initial body weights of the growing lambs were approximately equal among experimental groups. High saline water had adverse effect (*p* < 0.001) on the lambs’ average daily weight gains and final weights, as well as total live body weight gains, compared with LS lambs. Supplementing the HS lambs’ ration with protein afforded increase (*p* < 0.001) by 23.5% in daily body weight gains. Accordingly, the final body weight of HS lambs was improved (*p* < 0.001) 10.6% by protein supplementation. No differences for productive performance traits were detected between HS lambs supplemented with protein and the LS lambs fed the LP ration.

**Table 7:** Effects of protein supplementation and water salinity on productive performance of growing Barki lambs

	LP*		HP		SEM	*p* values		
	LS	HS	LS	HS		Protein	Water	P*W
Initial live body weight, kg	20.7	20.9	20.6	20.8	0.25	0.89	0.77	0.99
Final live body weight, kg	38.4^b^	34.6^c^	42.2^a^	38.7^b^	0.85	< 0.001	< 0.001	0.83
Total body weight gain, kg	17.7^b^	13.7^c^	21.6^a^	17.9^b^	0.88	< 0.001	< 0.001	0.83
Daily body weight gain, g	118^b^	91.4^c^	144^a^	119^b^	5.63	< 0.001	< 0.001	0.20
Feed conversion, kg/kg gain								
Dry matter	12.3^b^	14.8^a^	10.2^d^	11.4^c^	0.50	< 0.001	< 0.001	< 0.001
Total digestible nutrient	7.38^b^	8.29^a^	6.25^d^	6.80^c^	0.23	< 0.001	< 0.001	< 0.001
Digestible crude protein	0.67^b^	0.77^a^	0.74^ab^	0.77^a^	0.02	0.25	0.05	0.31

^*^LP, low crude protein ration (containing concentrate feed mixture CFM of 14% CP); *HP*, high crude protein ration (containing CFM of 20% CP); *LS*, low saline water (658 mg/L total dissolved solids); and *HS*, high saline water (2100 mg/L total dissolved solids). ^a,b,c,d^within a row, values with different superscript letters mean there were significant differences (*p* < 0.05)

Regarding feed conversion ratio (Table 7) in terms of the amount of DM and TDN used for producing one Kg gain, the best feed conversion values (*p* < 0.001) were recorded in protein supplemented groups as for LS lambs 10.2 and 6.25 kg/kg gain, respectively, followed by HS lambs 11.4 and 6.80 kg/kg gain, respectively. However, the worse feed conversion ratios were recorded for the HS lambs that fed LP ration. The value of feed conversion expressed as DCP intake/kg gain was better in LS lambs received LP ration without difference with LS lambs fed HP ration.

## Discussion

### Effect on water intake

The present research was designed to evaluate the effect of dietary protein supply to the requirements of growing lambs fed low-quality forage when drinking natural low or high saline water. Daily water intake of growing lambs increased with high saline water level (2100 mg TDS/L) by about 18% but was not significantly affected by protein supplementation. However, the increase of water intake occurred could be due to salt stimulates specific receptors on the tongue to activate osmoreceptors in the hypothalamus (Ghanem et al. 2018), as a physiological response to high salt ingestion for regaining an isotonic state (Enke et al. 2022). In Barki lambs, Ghanem et al. (2018) reported that the lambs accommodated to the water salty taste after the first month of the experiment and thus increased their water intake by the end of the experiment. In another study with Ouled Djellal ewes drank saline water containing 1 and 1.6% Nacl, the total water consumption increased in parallel with the increase in water salinity (Noureddine et al. 2022). Similarly, Yousfi and Salem (2017) observed a higher water intake in sheep offered saline water with 11 g and 15 g of NaCl compared with tap water group.

However, the present response to high saline water intake was also observed in goats (Mdletshe et al. 2017; Paiva et al. 2017; Zoidis and Hadjigeorgiou 2018; Runa et al. 2020; Costa et al. 2021). The goats water consumption increased by increasing the water total dissolved solid concentrations ranging from 640 to 8326 mg/L (Mdletshe et al. 2017; Paiva et al. 2017; Costa et al. 2021) and with increasing water NaCl concentrations until 1% (Zoidis and Hadjigeorgiou 2018). However, saline water increased goats water intakes by about 20% and 33% (young and old goats, respectively), compared with the experiment control phase (Runa et al. 2020). As demonstrated by Thiet et al. (2022), high water salinity affected the drinking behavior and increased the water consumption in Boer male crossbred goats.

### Effect on feed intake, nutrient digestibility, and rumen parameters

The present tested high saline water (2100 mg TDS/L) had adverse effect on dry matter intake and nutrients digestibility of growing lambs. This effect could be considered as response to increased high saline water intake. However, dry matter intake is one of the main important factors in the sheep production performance, being considered determinant for absorption of the nutrients required for animal maintenance and body gains (Araújo et al. 2019).

The data from the current study revealed that lambs consumed high saline water (2100 mg TDS/L) and fed CFM containing 20% CP had higher digestion of DM, OM and CP, as well as had greater growth performance compared to lambs consumed the high saline water and fed the CFM containing 14% CP. This positive response could be due to the higher feeding values and daily intakes of TDN and DCP in supplemented ration, meeting the requirements of the high saline water treated lambs. The existence of an interaction between high saline water and supplemental protein that alters the response of sheep to low-quality forage utilization was also confirmed by López et al. (2017).

In harmony with previous studies, the reduction in feed intake in animals that consumed high saline water was affected by a decrease in nutrient digestibility (Thiet et al. 2022). Protein supplementation is one of the strategies that improves the utilization of low-quality forages and nutrient digestibility and enhance the growth performance for animals fed low-quality forage (Obeidat et al. 2020; Shreck et al. 2021). Offering diets supplemented with protein improved the intake and passage rate of nutrients in response to the increase of the available nitrogen supply in the rumen (Obeidat et al. 2020). In this regard, López et al. (2017) stated that the total tract digestible organic matter increased approximately 30% in lambs consumed high saline water, when supplemented with dietary protein (0.25% BW/d of soybean meal).

Several studies indicated the role of saline water in feed intake and digestibility. Some studies have reported that feed intake is negatively affected by increasing levels of saline water. For example, Yousfi and Ben Salem (2017) reported that the administration of NaCl in water at rates of 11 and 15 g/L significantly decreased feed intake of Barbarine sheep by 4% and 14%, respectively, compared with tap water group. Katahdin sheep treated with brackish water source (5596 mg TDS /L) and higher levels of TDS through adding of NaCl observed a decreased feed intake and a linearly change in organic matter intake as total dissolved salts increased, while total tract organic matter digestion was not influenced (Yirga et al. 2018). Noureddine et al. (2022) observed that the salt level in the drinking water of Ouled Djellal ewes increased the water consumption, while the quantity of forage ingested decreased by 3–8%. In goats, high salinity in drinking water reported to decrease the dry matter intake (Mdletshe et al. 2017; Zoidis and Hadjigeorgiou 2018; Runa et al. 2019; Thiet et al. 2022). However, such previously mentioned discrepancies could be due to the variance of experimental breeds or animal species.

Conversely to the present results obtained, dry matter intake was not differed among treatments when sheep drank water with saline levels ranging from 640 to 8326 mg TDS/L (Moura et al., 2016). Moreover, the dietary supplementation with soybean up to 0.25% and 0.5% BW/d did not enhance the intake values of Hampshire lambs drinking high saline water with 8358 mg TDS/L (López et al. 2017). Furthermore, increasing water salinity concentration up to 8320 mg TDS/L did not affect intake of dry matter and nutritional fractions, nutrient digestibility, and water intake of Morada Nova sheep (Araújo et al. 2019) and Santa Ines sheep (Albuquerque et al. 2020). Similarly in goats, the same high level of saline water increased water intake, while it had no effect on the intake of dry matter, crude protein, neutral detergent fiber, ether extract, total digestible nutrients, total carbohydrates, non-fibrous carbohydrates, digestible energy, metabolizable energy, or apparent nutrient digestibility (Paiva et al. 2017). Likewise, Enke et al. (2022) reported that the increasing water intake was effective, while the reduction of feed intake was not detected.

In current study, the lambs’ ruminal pH and total VFA’s and ammonia–N concentrations were not affected by drinking the high saline water, while the ruminal ammonia–N increased as response to protein supplementation by about 36% and 21% for low or high saline water, respectively. Regardless of decreased rumen pH, Yousfi and Salem (Yousfi and Ben Salem 2017) revealed that the ammonia–N concentration in the rumen was not affected by the saline water consumption of Barbarine sheep. Otherwise, ruminal pH and ammonia–N concentration of Katahdin sheep was not affected by increasing water salinity consisting of brackish water and plus 6900 mg/l NaCl, while the total VFA concentration was decreased (Yirga et al. 2018).

### Effect on blood serum parameters

The current results revealed that high saline water had no effect on the lambs’ serum concentrations of total protein, albumin, globulin, albumin/globulin ratio, and creatinine, while it decreased the AST and ALT values and increased the urea-N. Supplementing the lambs exposed to the experiment high saline water with CFM containing 20% CP increased the serum total protein, ALT, AST, and urea-N concentrations, compared to lambs consumed high saline water and fed the CFM containing 14% CP. Protein supplementation enhanced the measured serum ALT and AST of lambs exposed to high saline water to be remained within the normal physiological ranges. This indicates remarkable adaptation capacities of growing lambs to present high saline water level without harmfully damage to liver and kidney functions.

However, serum biochemical values are critical indicators that can reliably assess the health status of animals. Serum ALT and AST are indicator enzymes of liver tasks to maintain the body’s metabolic homeostasis (Makawana et al. 2022). On another note, creatinine is a byproduct of muscle metabolism and is excreted by glomerular filtration; thus, it can be used as an indicator of renal function (Thiet et al. 2022). Moreover, blood urea is considering to be an indicator of ingested or mobilized protein (Pelegrin-Valls et al. 2020).

Previous studies evidenced the effect of high saline water on some blood biochemical parameters. In Barki sheep, saline water (4557 or 8934 ppm TDS), after 9 months of treatment, increased the AST, ALT, ALP, urea, and creatinine values, compared with tap water group (Ghanem et al. 2018). Saline water containing 11 or 15 g NaCl/l had no effect on the plasma total protein and albumin in Barbarine sheep, while the plasma urea was higher compared with those offered tap water, suggesting an alteration of kidney function (Yousfi and Ben Salem 2017). Moreover, the water salinity reported to increase blood protein levels and has no significant effect on the cholesterol, ALT, and AST while decreased creatinine and urea concentrations of Ouled Djellal sheep (Noureddine et al. 2022). On another hand, the increasing protein supplementation gradually increased the blood total protein and urea-N concentrations of growing lambs (Afroz et al. 2020). In this regard, plasma urea-N of Hampshire lambs treated with saline water (8358 mg TDS/L) linearly increased as the level of soybean meal supplementation increased (López et al. 2017). In goats, the increasing of water salinity reported to elevate the plasma concentrations of protein, creatinine, and urea levels (Zoidis and Hadjigeorgiou 2018). Runa et al. (2020) stated that high salinity water has no effect on plasma for ALT, AST, and urea, whereas the high salinity water increased plasma creatinine levels. Thiet et al. (2022) obtained that the creatinine concentration in plasma remained unchanged between the goat groups consuming highly saline water, indicating no adverse effects on kidney function.

### Effect on growth performance

High saline water caused significant adverse effect on average daily weight gain and final body weight of the current experiment growing lambs. Supplementing the lambs of high saline water with protein afforded increase by 23.5% in daily body weight gains; accordingly, their final body weight was improved by about 10.6%.

This present result is comparable to that obtained by Ghanem et al. (2018) as saline water treatment (4557 or 8934 ppm TDS) decreased the body weight of Barki lambs that was attributed to the decreased feed intake and/or metabolic failure. However, studies reported by Yousfi and Ben Salem (2017) pointed out a decrease in the body weight of Barbarine sheep receiving 1.5% and 2% NaCl in water. The decreased body weight of growing Ossimi lambs by drinking saline water (1.5% NaCl) and the deleterious effects on carcass characteristics and meat quality were detected by Hussein et al. (2020). These same authors reported that the betaine supplementation (2500 mg/kg concentrate mixture) improved the final weight by 7.47% of lambs consumed saline water compared with lambs consumed saline water without betaine supplementation. Similar negative effect of saline water on growth performance in goats was reported by Mdletshe et al. (2017) who detected that the weight gain was decreased with the increase of total solids dissolved in drinking water from 5.5 to 11 g TDS/L compared with the freshwater group. Thiet et al. (2022) stated that, in the 2nd week post-treatment, the weight gain tended to decrease in the goats drinking highly saline water, compared to those drinking freshwater. However, several studies attributed the lower weight gain of animals consumed high salinity water may be due to the lower dry matter intake (Mdletshe et al. 2017; Ghanem et al. 2018; Thiet et al. 2022).

Contrary to present results, no differences were observed in the average weight gain of Morada Nova sheep by consuming different water salinity levels up to 8320 mg TDS/L (Araújo et al. 2019). The goats’ body weight was not affected by saline water intake up to 1.5% NaCl, whereas body condition scores decreased (Runa et al. 2019, 2020). Enke et al. (2022) evaluated the sensitivity of llamas towards saline water with different NaCl concentrations up to 1.5% for 3 weeks; the authors found that the body weight and body condition scores were not negatively affected by the ingestion of saline water.

Supplementing the animal diet with crude protein is considered to be costly. Therefore, higher protein concentration as well as feed intake and digestibility can be reached by maintaining a balanced protein-to-energy ratio in the diet. Furthermore, this can reduce the animal metabolic discomfort and result in greater forage intake (Obeidat et al. 2020). In the present study, the used protein supplementation level greatly increased the corresponding value (as a price of life body weight gain) of growing lambs. The CFM containing 20% CP in lambs’ diet drunk high saline water caused higher return value and economic efficiency by 58.2% and 44.9%, respectively, than lambs’ drunk high saline water and fed CFM containing 14% CP. These higher economic efficiency values revealed that supplemented lambs’ group had the highest economical feed efficiency.

## Conclusions

This study demonstrated that the consumption of high saline water (2100 mg TDS/L) had negative effects on lambs’ low-quality forages intake, dry matter intake, nutrient digestibility, some blood parameters, and growth performance, when provided with diets containing 14% CP. These findings highlight the primordial role of water salinity as a limiting factor for small ruminants’ husbandry in high saline water resources. However, based on obtained positive results, the dietary protein supplementation is a beneficial tool to improve the performance and profitability of the lambs receiving 2100 mg TDS/L saline water with low-quality forages and thus offers livestock holders at semi-arid regions the possibility of using high saline water in the lambs feeding without deleterious effects. However, further studies are required to determine the growing lambs’ response with a supplemented protein diet to higher salinity levels of drinking water and to define the effect of water salinity and protein supplementation on meat and milk quality either for sheep or goats.

## Data Availability

The data obtained during the current study are presented in the manuscript and available from the corresponding author on reasonable request.
